# A latent class analysis of change and continuity in adolescent health and wellbeing in England during the decline in youth alcohol consumption: A repeat cross-sectional study

**DOI:** 10.1016/j.pmedr.2023.102481

**Published:** 2023-10-15

**Authors:** Abigail Kate Stevely, Laura A Gray, Hannah Fairbrother, Laura Fenton, Madeleine Henney, John Holmes

**Affiliations:** a Sheffield Alcohol Research Group, Sheffield Centre for Health and Related Research (SCHARR), School of Medicine and Population Health, University of Sheffield, UK; b Health Economics and Decision Science, Sheffield Centre for Health and Related Research (SCHARR), School of Medicine and Population Health, University of Sheffield, UK; c Health Sciences School, University of Sheffield, UK; d Healthy Lifespan Institute, University of Sheffield, UK

**Keywords:** Latent class analysis, HBSC, Trend analysis, Adolescent health, Health Behaviour in School-aged Children study, Alcohol Drinking, Underage Drinking, Young people

## Abstract

In England, the proportion of 13–15 year-olds who have ever drunk alcohol fell from 71% in 1999 to 35% in 2019. Despite substantial research literature studying this decline, we know little about connections with concurrent shifts in wider aspects of health and wellbeing. This paper aims to identify how indicators of health and wellbeing cluster within 15-year-olds in England, identify changes in clustering over time, and explore associations with sex and family affluence. We used latent class analysis of cross-sectional data from the Health Behaviours in School-aged Children study (n = 5,942; four waves 2001/02–2013/14). Classes were defined by indicators of substance use, sexual activity, diet, exercise, school-related measures, e-media use, parental relationships, and wellbeing. We identified three classes, which we labelled *Overall unhealthy*, *Substance abstainers with behavioural risk indicators*, and *Overall healthy*. The probability of being in the *Overall unhealthy* class fell (2001/02: 0.39; 2013/14: 0.18) while the probability of being in the *Overall healthy* class increased (0.21 to 0.41). The probability of weekly alcohol use fell in all classes (e.g. *Overall unhealthy*: 0.71 to 0.28). Females (female vs male OR: 1.74 95%CI: 1.30 – 2.34) and those with low family affluence (high vs low family affluence OR: 0.18 95%CI: 0.08 – 0.44) had significantly higher odds of being in the *Overall unhealthy* class. Overall, adolescents became more likely to have co-occurring indicators of good health and wellbeing, including reduced alcohol consumption, sexual activity and cigarette smoking. However, girls and those from poorer families remained more likely to have poor health and wellbeing.

## 1 Introduction

Since the early 2000s, there have been marked trends in multiple aspects of adolescent health and wellbeing in many high income countries, including declines in substance use (Vashishtha et al., 2020, De Looze et al., 2019, Vashishtha et al., 2020, Lewycka et al., 2018). For example, between 2000 and 2018 in England, the prevalence of cigarette smoking among 15-year-olds fell by 18 percentage points and illicit drug use by 10 percentage points (NHS Digital, 2022). Of these trends, declines in alcohol consumption have been particularly marked and have received considerable research attention (Vashishtha et al., 2020, Vashishtha et al., 2020). The proportion of 13–15 year-olds in England who have ever had an alcoholic drink has fallen from 71% in 1999 to 35% in 2019, with the decline in its entirety occurring between 2003 (74%) and 2014 (34%) (Lifestyles Team, 2019). Despite substantial international evidence that these trends are concurrent, we know little about the connections between them or their main drivers (Patalay and Gage, 2019, Hublet et al., 2015).

One approach to understanding the connections between health and wellbeing trends is to analyse changes over time in how they cluster within individuals. A recent systematic review found some evidence that health-related behaviours cluster within 11–16 year-olds in consistent ways across studies conducted in different times and places (Whitaker et al., 2021). Most primary studies identified a class of adolescents likely to engage in multiple ‘healthy’ behaviours and a class likely to engage in multiple ‘unhealthy’ behaviours, particularly use of alcohol, tobacco and illicit drugs. The remaining classes showed mixed behavioural patterns that varied across studies. Much less is known about how such clusters change within a population over time. The longitudinal or repeat cross-sectional datasets used by the primary studies were analysed largely either using only one wave of data or by pooling data from multiple waves. Changes in individual-level clustering are important for understanding changing adolescent health risk profiles, and the mechanisms driving concurrent trends across health and wellbeing indicators. For example, if members of an unhealthy class in cluster analyses becomes less likely to engage in cigarette use, illicit drug use and alcohol consumption over time but communication with parents changes only among members of other classes, this would suggest that declines in substance use may share common causes that are unrelated to changes in parental relationships.

The clustering of health and wellbeing indicators also has important implications for adolescent health inequalities, although there is little evidence available on this topic (Whitaker et al., 2021). Health-related behaviours differ between boys and girls and also play an important role in other health inequalities (Lewycka et al., 2018, Inchley et al., 2013). There is also evidence that lower socio-economic status is associated with a higher likelihood of engaging in multiple, as well as single, risk behaviours (Kipping et al., 2015). As such, changes in the clustering of health and wellbeing indicators may suggest shifts in distal social determinants of health inequalities rather than more proximal determinants. Alternatively, changes in patterns of clustering may contribute to health inequalities where they increase the concentration of disadvantaged groups within the least healthy clusters.

This paper is particularly interested in the changing position of alcohol consumption within such clusters as its analyses form part of a larger project investigating the international decline in youth drinking (Whitaker et al., 2021, Holmes et al., 2022). The existing quantitative evidence provides some support for a number of mechanisms that may have contributed to that decline, including changes in parenting, alcohol policy, and reductions in face-to-face socialising (Vashishtha et al., 2020). However, much of this literature focuses on testing single mechanisms in isolation and these explain only a small to moderate proportion of the trend (De Looze et al., 2019, Vashishtha et al., 2020). Overall, the evidence suggests that no single dominant mechanism drives the decline in youth drinking. Instead, it may be part of a wider set of changes shaping multiple aspects of adolescent health and wellbeing (Pennay et al., 2018, Caluzzi et al., 2020). Examining changes in the clustering of health and wellbeing indicators within individuals over time may therefore help to improve our understanding of recent trends in youth drinking. In particular, it may provide insights into whether the decline in alcohol consumption reflects alcohol becoming disconnected from other ‘problem behaviours’ associated with adolescence (Jessor, 1991), a general shift among adolescents away from such problem behaviours, or a combination of the two.

This paper will use latent class analysis to examine: (i) how indicators of adolescent health and wellbeing cluster within 15-year-olds in England; (ii) how the prevalence of each cluster and the presence of each indicator within those clusters changes over time and (iii) how associations change over time between the clusters and two domains of health inequalities: sex and family affluence.

## 2 Methods

### 2.1 Data

The Health Behaviours in School-aged Children study (HBSC) is an international repeat cross-sectional survey with measures covering health and wellbeing (Inchley et al., 2013). Within each participating country, HBSC uses a clustered sampling design to recruit schoolchildren aged 11-, 13-, and 15-years-old, with a target sample size of 1500 respondents per age group. In England, a random sample of individuals is selected from a sample of all secondary schools, stratified by region and type (i.e. state or independent). Data is collected using questionnaires administered under exam conditions by either teachers or members of the research team (Magnusson et al., 2015).

We analysed English data from the 2001/02, 2005/06, 2009/10 and 2013/14 waves, which coincide with the decline in youth drinking (Inchley et al., 2013). Our analytical sample included only 15-year-olds because the prevalence of indicators of interest - including alcohol consumption - is very low at younger ages. The total analytical sample size was 5,942 participants.

### 2.2 Measures

#### 2.2.1 Measure selection process

The HBSC survey includes a wide range of self-reported adolescent health and wellbeing indicators, including behaviours, attitudes, life satisfaction and relationships. Since latent class models can be highly sensitive to the measures included, we used a structured approach to selecting the most appropriate measures for our research aims. This approach was informed partly by our interest in the decline in adolescent drinking and we selected measures that could provide insights into this. First, the research team reviewed the available measures across all four waves of the survey and developed a set of candidate measures based on which survey questions and response options were consistent over time and their relevance to hypothesised explanations for the decline in adolescent drinking. For example, ease of communication with parents is relevant to hypotheses that posit adolescents having closer relationships with parents and spending more time at home caused the decline (Törrönen et al., 2019). We considered a wide range of contemporary indicators of health and wellbeing including e-media use, which is associated with alcohol consumption and mental wellbeing, and is a persistent area of concern related to various aspects of adolescent health and wellbeing, even if the nature of these relationships remains unclear (Walsh et al., 2020).

Second, we conducted an online consultation with a purposive sample of academic and non-academic stakeholders to ensure our findings would be relevant to researchers and public health practitioners in the field. This involved eliciting feedback on whether any of the candidate measures should be excluded and whether any additional measures should be included. Third, the research team discussed the feedback and agreed a final set of variables for use in the analyses, which are described below and in Supplementary Table S1 alongside rationales for each measure. We excluded one of our candidate measures of dietary behaviour - soft drink consumption - following stakeholder advice that adolescents do not use soft drinks as a substitute for alcohol consumption.

#### 2.2.2 Measures used to identify classes

Our final set of health and wellbeing indicators is fully described in Supplementary Table S1. We used binary or ordinal measures of substance use, wider health-related behaviours, education-related factors, relationships, ease of communication with parents, and wellbeing. The substance use measures were weekly alcohol use (weekly drinker vs not), cigarette smoking (current smoker vs non-smoker) and lifetime cannabis use (any cannabis use vs none). Wider health-related behaviours were exercise (four levels, based on number of days participants were active in the last week), fruit and vegetable consumption (four levels, based on how many times a week participants usually eat fruit and vegetables), and sexual activity (had sexual intercourse vs never). Education-related factors were perceived academic achievement (four levels, based on the participant’s opinion on what their class teacher thinks about their performance in class) and feeling pressured by schoolwork (four levels from feeling a lot of pressure, to not at all). Relationship measures were classmate social support (four levels, based on subscales measuring (i) students enjoy being together (ii) are kind and helpful, (iii) accept me as I am), e-media use (daily user vs not), and ease of communication with parents (binary, whether the participant has one or more parents/step-parents who are very easy to talk to). Wellbeing was measured using life satisfaction (four levels, based on self-rated best possible life to worst possible life).

#### 2.2.3 Socio-demographic predictors

We used measures of family affluence and sex as independent predictors of class membership (Supplementary Table S1). Family affluence is measured using the Family Affluence Scale, which is based on questions assessing car/van/truck ownership, adolescents having their own bedroom, family holidays, and computer ownership (Inchley et al., 2013). Sex is a self-reported dichotomous measure (male/female).

### 2.3 Statistical analysis

The analysis had four stages. First, we conducted descriptive analyses of socio-demographic characteristics and trends in health and wellbeing indicators. Second, we used multiple imputation to prepare the dataset for analysis. Third, we selected the best-fitting model specification for the primary analysis. Fourth, we used the best-fitting model specification to estimate LCA models for the primary analysis and for three sensitivity analyses. The detailed methods description below focuses on stages two to four. We used Stata v16 for descriptive analysis and MPLUS version 8.6 for multiple imputation and latent class analyses.

#### 2.3.1 Multiple imputation

MPLUS removes observations with missing data on covariates from the analysis. To minimise the number of removed observations, we used multiple imputation across all four pooled cross-sectional survey waves via the MPLUS command *impute* to estimate values where family affluence or participant sex was missing. We generated 50 imputed datasets using the health and wellbeing indicators for the primary analysis as predictors. Family affluence and participant sex were also included as predictors in the imputation for each other.

#### 2.3.2 Selecting the best-fitting model

We then analysed the imputed datasets to identify latent class models with between two and six classes, using all four pooled cross-sectional survey waves. When analysing a set of imputed datasets, MPLUS models each dataset separately and outputs average model parameters. The models were adjusted for clustering of individuals within schools, weighted using HBSC sample weights (Currie et al., 2014), and included family affluence and participant sex as covariates. We used 1000 random starts for each model and checked that the lowest log-likelihood value was replicated to avoid local minima. We selected the preferred number of latent classes using the adjusted-BIC and a qualitative assessment of class separation (i.e. whether the classes can be interpreted as distinct and meaningful) (Weller et al., 2020).

#### 2.3.3 Estimating the latent class models

After selecting the number of classes, we then sought to identify the model that best captured the observed changes over time. We compared a series of models with specifications that allowed different levels of variation in estimated parameters across the four survey waves. Survey year was included in the models as a grouping variable and the relationship between the covariates and the probability of class membership was held constant across years. This is a recognised approach to fitting multiple group latent class models which tests for differences in class structure and size over time (Geiser et al., 2010). We initially estimated three model specifications:

1. Fully unconstrained: both the probabilities of class membership (the likelihood of individuals being in each class), and the conditional response probabilities (e.g., the likelihood of participants in each class being weekly drinkers) could vary across years;
2. Semi-constrained: only the probabilities of class membership could vary across years;
3. Fully constrained: class membership and conditional response probabilities were constant across years;

We then selected the best-fitting model specification based on a comparison of the adjusted-BIC values for these three models. Next we compared the best-fitting model with an alternative specification that allowed the relationship between the covariates and the probability of class membership to vary over time, again selecting the best-fitting model based on the adjusted-BIC.

MPLUS does not provide conditional response probabilities when analysing a set of imputed datasets, although it does provide average class membership probabilities and parameters for the relationship between covariates. We therefore generated the final conditional response probabilities by applying fixed model parameters from the final model to the first imputed dataset.

#### 2.3.4 Sensitivity analyses

Three sensitivity analyses were conducted to assess the robustness of the final model to the selection of alternative indicator variables. The sensitivity analyses used the same model specification as the primary analysis while separately replacing: (i) the primary measure of weekly drinking with an alternative measure of the same concept that includes alcopops (i.e. pre-mixed spirits) consumption from 2005/06 onwards; (ii) weekly drinking with lifetime drunkenness and (iii) pressure from schoolwork with liking school (Supplementary Table S1).

Ethical approval

This study was approved by the University of Sheffield’s ethics committee (040084) and conforms to the principles embodied in the Declaration of Helsinki. Each country that participated in the HBSC study obtained approval to conduct the survey from their ethics review board or equivalent regulatory body. Participation was voluntary, and informed consent (active or passive) was sought from school administrators, parents and children as per national human subject requirements (Magnusson et al., 2015).

### 2.4 Role of the funding source

The study sponsors had no role in study design; in the collection, analysis, and interpretation of data; in the writing of the report; or in the decision to submit the paper for publication.

## 3 Results

### 3.1 Socio-demographic characteristics and trends in health and wellbeing indicators

The weighted analytical sample was balanced by sex across all survey years (Supplementary Table S2). There were fewer participants with low than with middle or high family affluence, and this proportion also fell over time, particularly between 2001/02 and 2005/06 (Supplementary Table S2).

Time trends varied across health and wellbeing indicators (Table 1). The largest changes in prevalence between 2001/02 and 2013/14 were falls in weekly alcohol use (46% to 8%), cigarette smoking (32% to 12%), sexual activity (38% to 20%) and cannabis use (40% to 19%). There was also an increase in the proportion of participants reporting daily e-media use, from 45% in 2001/02 to 76% in 2013/14.

**Table 1:** Descriptive trends in health and wellbeing indicators among adolescents in England between 2001/02 and 2013/14.

	**Participants with missing data**	**Participants by survey year**							
	**All years**	**2001/02**		**2005/06**		**2009/10**		**2013/14**	
	**N**	**N**	**%**	**N**	**%**	**N**	**%**	**N**	**%**
**Weekly alcohol use**	68								
Not weekly		938	54	974	68	918	81	1456	92
Weekly		813	46	467	32	176	19	132	8
**Cigarette smoking**	41								
Non-smoker		1199	68	1110	77	912	81	1407	88
Smoker		558	32	339	23	190	19	186	12
**Sexual activity**	266								
Never		1051	62	998	71	773	71	1215	80
Had intercourse		649	38	404	29	277	29	309	20
**Lifetime cannabis use**	173								
Never		1016	60	1055	76	860	77	1271	81
Any use		683	40	337	24	240	23	307	19
**Perceived academic achievement**	143								
Very good		323	18	293	21	236	21	426	27
Good		745	42	626	45	533	49	722	46
Average		567	32	373	27	289	27	351	23
Below average		128	7	94	7	39	4	54	3
**Pressure from school work**	131								
Not at all		127	7	76	6	78	7	98	6
A little		488	28	403	29	369	34	500	31
Some		560	32	408	30	334	30	522	33
A lot		581	33	483	35	315	28	469	30
**Classmate support scale**	127								
High support		251	14	551	40	213	18	389	25
–		398	23	328	24	315	29	378	24
–		656	37	310	22	345	32	441	28
Low support		454	26	190	14	218	21	378	24
**Daily use of e-media**	226								
No daily use		960	55	611	43	368	33	366	24
Daily use		807	45	807	57	660	67	1137	76
**Ease of communication with parents**	164								
One parent is easy to talk to		757	43	604	42	389	36	669	44
None easy to talk to		1009	57	834	58	665	64	851	56
**Exercise**	134								
High		287	16	193	13	177	18	216	14
–		410	24	341	24	244	25	382	24
–		532	30	486	34	368	35	539	34
Low		524	30	411	29	264	21	434	28
**Fruit and vegetable consumption index**	45								
High		311	17	444	31	299	28	435	27
–		373	21	335	23	242	23	383	24
–		500	28	364	25	293	27	463	29
Low		582	33	301	21	263	22	309	19
**Life satisfaction**	161								
High		315	18	341	24	203	20	235	15
–		449	26	412	29	303	30	399	25
–		396	23	308	21	267	25	377	24
Low		535	32	378	26	304	26	559	36
**Lifetime drunkenness**	44								
None		524	30	538	37	556	45	924	58
Any lifetime drunkenness		1243	70	912	63	536	55	665	42
**Liking school**	94								
I like it a lot		269	15	345	25	187	15	305	19
I like it a bit		775	44	694	50	606	56	835	53
I don’t like it very much		469	26	230	17	208	19	313	20
I don’t like it at all		257	15	124	9	100	10	131	8

N = Unweighted number of participants in the Health Behaviours in School-aged Children study. % = Weighted percentage of participants.

### 3.2 Model fitting

We found diminishing improvements in the adjusted-BIC beyond three classes (Supplementary Table S3), and difficulties in model convergence beyond four classes. Qualitative assessment of class separation determined that the three-class model was the most clearly interpretable solution (Weller et al., 2020). The primary analyses therefore used a three-class model. The results for the four-class model are available in Supplementary Table S4 for comparison.

The fully unconstrained three-class model with constrained covariate parameters had the lowest adjusted-BIC. This means the final model allowed both the probabilities of class membership and the conditional response probabilities to vary across years, but held constant the relationships between sex, family affluence and class membership (Supplementary Table S5).

### 3.3 Primary analysis

**Fig. 1:**
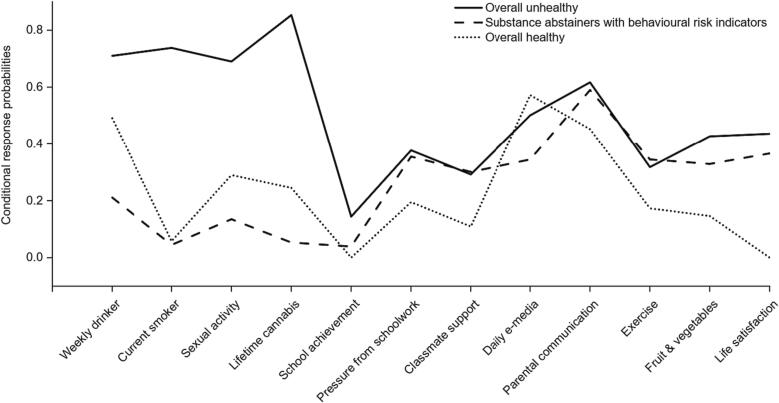
Conditional response probabilities in 2001/02 for adolescents in England in each latent class *These figures show the probability that a member of each class in a given year endorses the *most risky/unhealthy* category for each indicator. For some indicators, there is no clear risky or unhealthy category. For these we have selected based on interpretability, and all categories chosen are indicated in Supplementary Table 6.

The final model described three types of 15-year-olds in England, based on indicators of their health and wellbeing. In line with Whitaker et al., we labelled these *Overall unhealthy*, *Substance abstainers with behavioural risk indicators (BRIs)*, and *Overall healthy* (Whitaker et al., 2021). These labels are based on the LCA results for all four survey waves but we focus initially on the 2001/02 results to introduce each class (Fig. 1, Supplementary Table S6). All figures present conditional response probabilities for the least ‘healthy’ or lowest wellbeing category of each variable, as indicated in Supplementary Table S6.

**Fig. 2:**
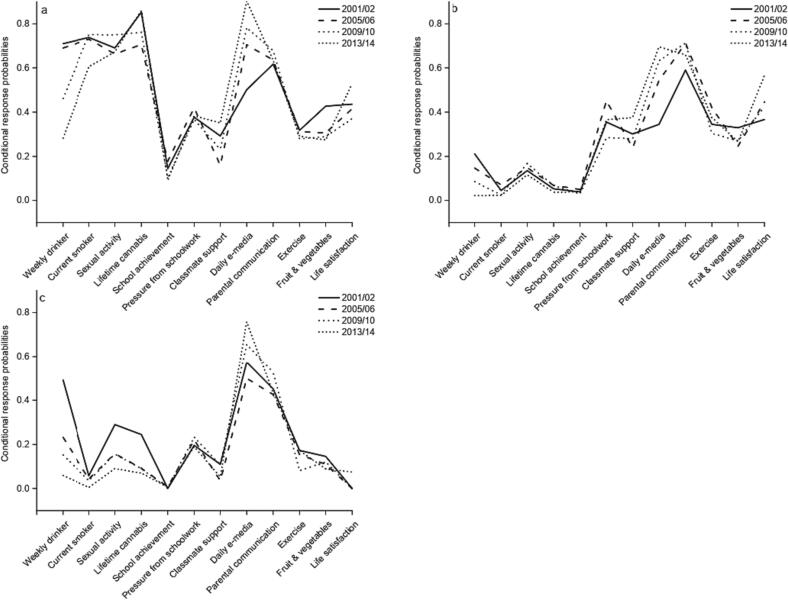
Conditional response probabilities over time for adolescents in England in (a) the Overall unhealthy class (b) the Substance abstainers with behavioural risk indicators class (c) the Overall healthy class*These figures show the probability that a member of each class in a given year endorses the *most risky/unhealthy* category for each indicator. For some indicators, there is no clear risky or unhealthy category. For these we have selected based on interpretability, and all categories chosen are indicated in Supplementary Table 6.

Fig. 1 and Supplementary Table S6 present the best-fitting latent class model for 2001/02. The *Overall unhealthy* class comprised 39% of the population in 2001/02 and had the highest conditional response probabilities (CRP) for most indicators of poor health or wellbeing, including weekly drinking (CRP = 0.71), current smoking (CRP = 0.74), and lifetime sexual activity (CRP = 0.69). They were, however, similar to the *Substance abstainers with BRIs* class on pressure from schoolwork, classmate support, ease of communication with parents and exercise. The *Substance abstainers with BRIs* class comprised 40% of the population in 2001/02 and differed from the *Overall unhealthy* class as they had the lowest prevalence of weekly drinking (CRP = 0.21), current smoking (CRP = 0.06), lifetime sexual activity (CRP = 0.14) and lifetime cannabis use (CRP = 0.05). The *Overall healthy* class comprised 21% of the population in 2001/02 and, despite their label, were moderately likely to report weekly drinking (CRP = 0.49), lifetime sexual activity (CRP = 0.29) and lifetime cannabis use (CRP = 0.25), although this changed over time (Fig. 2). Like the *Substance abstainers with BRIs* class, they had a low prevalence of current smoking (CRP = 0.06). Participants in the *Overall healthy* class were the least likely to report poor outcomes for school-related measures, ease of parental communication, exercise, fruit and vegetable consumption, and life satisfaction.

**Fig. 3:**
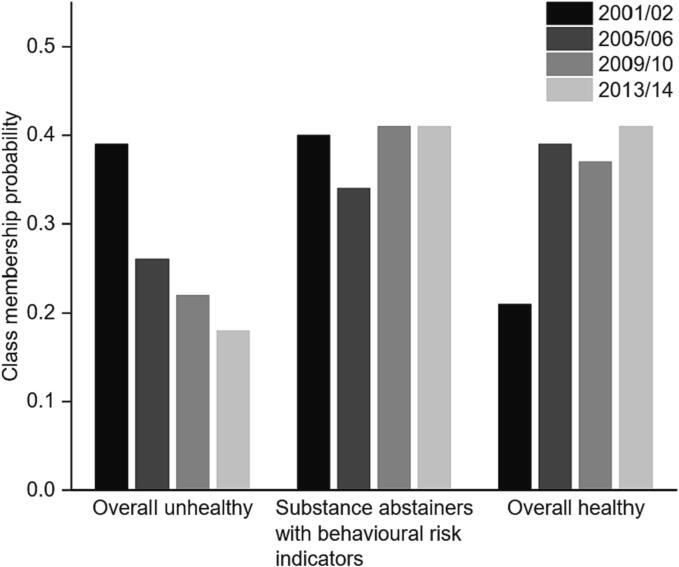
Latent class membership probabilities over time among adolescents in England*These figures show the probability that a member of each class in a given year endorses the *most risky/unhealthy* category for each indicator. For some indicators, there is no clear risky or unhealthy category. For these we have selected based on interpretability, and all categories chosen are indicated in Supplementary Table 6.

Fig. 3 shows the class membership probabilities over time. The probability of membership of the *Overall unhealthy* class fell from 39% in 2001/02 to 18% in 2013/14, with the largest change between 2001/02 and 2005/06. The probability of membership of the *Substance abstainers with BRIs* class was relatively consistent over time, ranging between 34% and 41%, while the probability of membership for the *Overall unhealthy* class increased from 21% in 2001/02 to 41% in 2013/14, with the largest change again between 2001/02 and 2005/06.

The conditional response probabilities within each class also changed over time (Fig. 2, Supplementary Table S7). Weekly alcohol use and smoking became less common in all classes over time. The magnitude of this change was greatest for alcohol, with CRPs declining between 2001/02 and 2013/14 from 0.71 to 0.28 in the *Overall unhealthy* class, from 0.21 to 0.02 in the *Substance abstainers with BRIs* class and from 0.49 to 0.06 in the *Overall healthy* class. Trends differed for other indicators and were not always consistent across classes, with life satisfaction worsening in both the *Overall healthy* class and the *Substance abstainers with BRIs* classes, the probability of reporting daily e-media use increasing over time in all classes, and no clear trends in exercise. The three classes remained identifiably similar over time, suggesting that there was no disappearance of old classes or emergence of new classes.

The best-fitting model indicated the association between both sex and family affluence, and class membership did not change over time. Table 2 presents odds ratios of class membership by sex and family affluence, which can be interpreted in a similar way to a multinomial logistic regression. Girls had significantly higher odds than boys of being in both the *Overall unhealthy* or *Substance abstainers with BRIs* classes compared to the *Overall healthy* class. Those with low family affluence were more likely to be in both the *Overall unhealthy* and *Substance abstainers with BRIs* than those with middle or high family affluence.

**Table 2:** Odds of latent class membership among adolescents in England between 2001/02 and 2013/14 by participant sex and family affluence.

	Odds of being in class vs. *Overall healthy* (95% confidence interval)	
	*Overall unhealthy*	*Substance abstainers with behavioural risk indicators*
Odds ratio for female vs male participant sex	1.74 (1.30–2.34)	1.88 (1.06–3.35)
Odds ratio for middle vs low family affluence	0.35 (0.17–0.75)	0.43 (0.19–0.97)
Odds ratio for high vs low family affluence	0.18 (0.08–0.44)	0.14 (0.06–0.33)

### 3.4 Sensitivity analysis

The sensitivity analysis results are consistent with the main results from the primary analysis (Supplementary Table S8-S10).

## 4 Discussion

We identified three classes of adolescents in England: *Overall unhealthy*, *Substance abstainers with behavioural risk indicators*, and *Overall healthy*. We highlight three main findings from our examination of these classes. First, the likelihood of adolescents being in the *Overall unhealthy* class fell, while the likelihood of being in the *Overall healthy* class increased over time, particularly between 2001/02 and 2005/06. Second, the prevalence of some health and well-being indicators (e.g. diet, physical activity, perceived academic achievement) remained largely stable in each class, suggesting any improvements in these indicators in descriptive trends are part of a broad shift towards improved adolescent health, rather than being a phenomenon specific to particular health risk factors. Other indicators, notably alcohol use, became less prevalent in all classes, suggesting additional alcohol-specific factors may drive trends. Moreover, it suggests the risk profile of adolescents is improving even within those we class as *Overall unhealthy*. Conversely, some indicators became more prevalent in some or all classes, suggesting aspects of adolescent risk profiles are also worsening beneath the general improvements. This includes reductions in life satisfaction in the intermediate *Substance abstainers with BRIs* class, and increases in daily e-media use in all classes, although the extent to which frequent e-media use indicates better or worse wellbeing is unclear (Shankleman et al., 2021). Third, female sex and lower family affluence were consistently associated with a higher likelihood of being in the *Overall unhealthy* class. This suggests persistent inequalities underlie the overall improvements in adolescent health and wellbeing.

Our analysis was informed by an interest in the international decline in adolescent alcohol consumption. In the absence of evidence for a single dominant driver of this decline, the findings above provide support for arguments that it is instead driven by multiple intersecting mechanisms that underlie or interact with broader shifts in adolescent health and wellbeing (Törrönen et al., 2019, Townshend, 2013). The substantial decline in alcohol consumption *within* all of the identified latent classes over time suggests that, even within a broader shift away from risk-related behaviours among adolescents, the shift away from alcohol is particularly pronounced. This perhaps reflects the much earlier emergence of negative attitudes towards smoking and, conversely, some adolescents’ perspective that cannabis use is low-risk (Whitaker et al., 2023). Future research should seek to further unpick this distinct status of alcohol within wider shifts in adolescent health and wellbeing.

One further finding merits additional attention. We add to previous evidence that frequent users of e-media have higher levels of alcohol consumption (De Looze et al., 2019, Ng Fat et al., 2021, Vashishtha et al., 2022). This suggests that any link between increases in adolescent e-media use and the decline in drinking is not a straightforward causal relationship (Vashishtha et al., 2020, Törrönen et al., 2019). Trends in e-media and alcohol use may instead be connected to broader shifts in young people’s lives driven by upstream determinants of health. Furthermore, quantitative literature in this area often uses simple measures of e-media use, and more nuanced approaches may be needed to unpack complex relationships with alcohol (Caluzzi et al., 2022).

Key strengths of this study include the use of a wide range of indicators of health and wellbeing that we selected in consultation with a group of expert stakeholders. We used data from four waves of the Health Behaviours in School-aged Children (HBSC) study, which is a large, nationally-representative dataset. The HBSC methodology has been described in detail by the study team, and variables have been carefully selected (Currie et al., 2014). The main limitations of our analysis are that some factors of interest to our expert stakeholders were not available in the dataset, such as adolescents’ exam grades. Also, although the decline in youth drinking largely occurred during the time period we analysed, the clustering of health and wellbeing indicators is likely to have changed since 2013/14, especially given changes in the nature and prevalence of e-media use and the COVID-19 pandemic. Future research using more recent data may be useful, as well as similar analysis using data from other countries which have seen a decline in youth drinking.

Our findings suggest that general improvements in adolescent health and wellbeing in England are likely to lead to significant immediate improvements and may also produce longer-term improvements in public health. For example, hospital admissions for violent, self-inflicted and drug-related injuries among adolescents fell in England between 2005 and 2011 (Herbert et al., 2017). If improvements in health and well-being indicators persist as this generation moves into adulthood, this may reduce future rates of chronic health conditions, particularly those related to alcohol (Holmes et al., 2022). However, the persistence of inequalities across the wide range of health and wellbeing indicators studied is concerning, particularly given that the alcohol-related harm experienced by disadvantaged groups is disproportionate to their consumption (Boyd et al., 2022, Holmes et al., 2016, Zaborskis and Grincaite, 2018). Given our findings suggest that trends in adolescent health and wellbeing are interconnected, it may be more effective and cost-effective to address the upstream social determinants of health that drive many of these indicators rather than targeting each indicator separately through behavioural and other interventions (Jessiman et al., 2021). Interventions with the potential to reduce such entrenched inequalities may need to be cross-sectoral, such as adopting a Health in All Policies approach which considers the health implications of all public policy decisions and seeks to improve public health and health equity (World Health Organization. Health in All Policies (HiAP) Framework for Country Action., 2014).

## Declaration of Competing Interest

The authors declare that they have no known competing financial interests or personal relationships that could have appeared to influence the work reported in this paper.

## Data Availability

The data used is available from https://hbsc.org/
