# Supplementary material for: A latent class analysis of change and continuity in adolescent health and wellbeing in England during the decline in youth alcohol consumption: A repeat cross-sectional study
Source: Prev Med Rep. 2023 Oct 15;36:102481. doi: 10.1016/j.pmedr.2023.102481 (PMC10594568; doi:10.1016/j.pmedr.2023.102481)
Supplement: Supplementary data 1 [file mmc1.docx]

**Supplementary material**

[Supplementary Table 1. Description of variables used to identify classes (indicators) and to predict class membership (covariates) 2](#_Toc118880108)

[Supplementary Table 2. Descriptive socio-demographic characteristics 7](#_Toc118880109)

[Supplementary Table 3. Selecting number of latent classes using adjusted-BIC 8](#_Toc118880110)

[Supplementary Table 4. Item response probabilities for the four class model 9](#_Toc118880111)

[Supplementary Table 5. Selecting model specification using adjusted-BIC 11](#_Toc118880112)

[Supplementary Table 6. Class membership and item response probabilities for the primary analysis in 2001/02 12](#_Toc118880113)

[Supplementary Table 7. Item response probabilities for the primary analysis 14](#_Toc118880114)

[Supplementary Table 8. Item response probabilities for the lifetime drunkenness sensitivity analysis 16](#_Toc118880115)

[Supplementary Table 9. Item response probabilities for the weekly drinking with alcopops sensitivity analysis 18](#_Toc118880116)

[Supplementary Table 10. Item response probabilities for the liking school sensitivity analysis 20](#_Toc118880117)

# Supplementary Table 1. Description of variables used to identify classes (indicators) and to predict class membership (covariates)

| **Indicators for primary analysis** | **Rationale** |
| --- | --- |
| **Weekly alcohol use** |  |
| At present, how often do you drink anything alcoholic, such as beer, wine or spirits? Try to include even those times when you only drink a small amount. A. Beer B. Wine C. Spirits  Every day; every week; every month; rarely; never  Classified as a weekly drinker if they drank any of beer, wine or spirits every week or every day.  0 Not a weekly drinker  1 Weekly drinker* | We have selected a binary measure of weekly alcohol use for the primary analysis, as it captures current drinking behaviour.  Using a binary measure facilitates combining measures of drinking frequency across different beverage types. |
| **Cigarette smoking** |  |
| How often do you smoke tobacco at present?  Every day; at least once a week, but not every day; less than once a week; I do not smoke  Classified as a smoker if they reported any response other than ‘I do not smoke’.  0 Non-smoker  1 Smoker* | Cigarette smoking is a key substance use behaviour, and is therefore relevant for our analysis.  We used a binary measure of current smoking status because smoking is a minority behaviour within our population. If we split participants into light and heavy smokers, this would result in a very small group and is unlikely to be meaningful in cluster analysis. |
| **Sexual activity** |  |
| Have you ever had sexual intercourse (sometimes this is called “making love”, “having sex”, or “going all the way”)?  Yes; no  0 Never had sexual intercourse  1 Had sexual intercourse* | We included sexual activity as an indicator of transition to adult behaviour. Stakeholders also thought that early sexual activity may be associated with substance use behaviours. |
| **Lifetime cannabis use** |  |
| Have you ever taken cannabis? A. In your life  Never; once or twice; 3 to 5 times; 6 to 9 times; 10 to 19 times; 20 to 39 times; 40 times or more  Classified as having used cannabis at least once in their lifetime if they reported any response other than ‘Never’.  0 Never used cannabis  1 Any cannabis use* | Cannabis use is a key substance use behaviour, and is therefore relevant for our analysis.  We used a binary measure of lifetime cannabis use because cannabis use is a minority behaviour within our population. If we split participants into light and heavy users, this would result in a very small group and is unlikely to be meaningful in cluster analysis. |
| **Perceived academic achievement** |  |
| In your opinion, what does your class teacher(s) think about your school performance compared to your classmates?  1 Very good  2 Good  3 Average  4 Below average* | We were interested in measures relating to education as increasing commitment to education and concern about employment prospects is one potential driver of the decline in adolescent drinking.  We chose perceived academic achievement as one of our primary measures in this area. |
| **Pressure from school work** |  |
| How pressured do you feel by the schoolwork you have to do?  1 Not at all  2 A little  3 Some  4 A lot* | We were interested in measures relating to education as increasing commitment to education and concern about employment prospects is one potential driver of the decline in adolescent drinking.  We chose pressure from school work as one of our primary measures in this area. |
| **Classmate support scale** |  |
| Here are some statements about the students in your class(es). Please show how much you agree or disagree with each one.   1. The students in my class(es) enjoy being together 2. Most of the students in my class(es) are kind and helpful 3. Other students accept me as I am   Strongly agree (1); agree (2); neither agree nor disagree (3); disagree (4); strongly disagree (5)  Responses to A. B. and C. are summed to create the Classmate support scale ranging from 3 to 15. We split this scale into quartiles based on the distribution of participant responses.  1 High support (3-5)  2 (6)  3 (7-8)  4 Low support (9-15)* | We were interested in the relationship between social support and alcohol use, so we included the classmate support scale.  The full scale includes a large number of categories, which we split into quartiles. This approach was chosen as a balance between information retention and maintaining a feasible contingency table for analysis. |
| **Daily use of e-media** |  |
| In the first three survey waves:  How often do you talk to your friend(s) on the phone or send them text messages or have contract through the internet?  Rarely or never; 1 or 2 days a week; 3 or 4 days a week; 5 or 6 days a week; every day  In the 2013/14 wave, this question was replaced with a series of questions:   - How often do you talk to your friends on the phone or internet based programmes such as Face Time or Skype? - How often do you contact your friends using texting/SMS? - How often do you contact your friends using email? - How often do you actively contact your friends using instant messaging (e.g. BBM, Facebook chat)? - How often do you contact your friends using other social media, such as Facebook (posting on wall, not chat), My Space, Twitter, Apps (e.g. Instagram), games (e.g. Xbox), YouTube, etc?   Hardly ever or never; less than weekly; weekly; daily  Classified as daily users in the first three waves if they responded ‘every day’. Classified as daily users in 2013/14 if they responded ‘daily’ to any of the five questions. Classified as non-daily users otherwise.  0 Not a daily user of e-media  1 Daily use of e-media* | Increasing use of e-media, including social media, has been hypothesised as a driver of the decline in adolescent drinking.  The available measures were inconsistent over time. We therefore constructed a new measure to identify daily use across all four survey waves. |
| **Ease of communication with parents** |  |
| How easy is it for you to talk to the following persons about things that really bother you? A. Father B. Stepfather C. Mother D. Stepmother  Very easy; easy; difficult; very difficult; don’t have or see this person  0 Has one or more parent(s) or step-parent(s) who is/are very easy to talk to  1 Has no parents or step-parents who are very easy to talk to* | Ease of communication with parents was selected as the best available measure of relationships with parents. Closer relationships with parents has been hypothesised as a driver of the decline in adolescent drinking.  We combined measures of communication with parents and step-parents to capture complex family structures. |
| **Exercise** |  |
| Physical activity is any activity that increases your heart rate and makes you get out of breath some of the time. Physical activity can be done in sports, school activities, playing with friends, or walking to school. Some examples of physical activity are running, brisk walking, rollerblading, biking, dancing, skateboarding, swimming, soccer, basketball, football, & surfing. For this next question, add up all the time you spent in physical activity each day.  Over the past 7 days, on how many days were you physically active for a total of at least 60 minutes per day?  0 days; 1 day; 2 days; 3 days; 4 days; 5 days; 6 days; 7 days  We split this scale into quartiles based on the distribution of participant responses.  1 High physical activity (7)  2 (5-6)  3 (3-4)  4 Low physical activity (0-2)* | Physical activity is a key health behaviour, and is therefore relevant for our analysis.  We selected this question as it is the only available measure that is consistent over time.  The full set of response options include a large number of categories, which we split into quartiles. This approach was chosen as a balance between information retention and maintaining a feasible contingency table for analysis. |
| **Fruit and vegetable consumption index** |  |
| How many times a week do you usually eat or drink…? A. Fruit B. Vegetables  Never (0); Less than once a week (0.25); Once a week (1); 2-4 days a week (3); 5-6 days a week (5.5); Once a day , every day (7); Every day, more than once (14)  Responses to A. and B. are summed to create a scale ranging from 0 to 28. We split this scale into quartiles based on the distribution of participant responses.  1 High fruit and vegetable consumption (17-28)  2 (10-15)  3 (6-8.5)  4 Low fruit and vegetable consumption (0-5.75)* | Diet is a key health behaviour, and is therefore relevant for our analysis.  There are a number of measures of dietary health in the survey, so in addition to consulting with our advisory group, we consulted with Dr Samantha Caton (a biological Psychologist with an interest in obesity and nutrition). We selected a combined fruit and vegetable index as the single best measure.  The full index includes a large number of categories, which we split into quartiles. This approach was chosen as a balance between information retention and maintaining a feasible contingency table for analysis. |
| **Life satisfaction** |  |
| Here is a picture of a ladder. The top of the ladder ‘10’ is the best possible life for you and the bottom ‘0’ is the worst possible life for you. In general, where on the ladder do you feel you stand at the moment?  We split this scale into quartiles based on the distribution of participant responses.  1 High life satisfaction (9-10)  2 (8)  3 (7)  4 Low life satisfaction (0-6)* | We selected self-rated life satisfaction as the best measure of general wellbeing.  The full set of response options include a large number of categories, which we split into quartiles. This approach was chosen as a balance between information retention and maintaining a feasible contingency table for analysis. |
| **Alternative indicators for sensitivity analyses** | **Rationale** |
| **Weekly alcohol use** |  |
| In the 2005/06 wave, D. Alcopops was added to the question above.  Classified as a weekly drinker if they drank any of beer, wine, spirits or alcopops every week or every day.  0 Not a weekly drinker  1 Weekly drinker* | Since alcopops were not included in all survey waves, we constructed a second measure of weekly drinking to be used in sensitivity analysis. |
| **Lifetime drunkenness** |  |
| Have you ever had so much alcohol that you were really drunk?  No, never; yes, once; Yes, 2-3 times; Yes, 4-10 times; Yes, more than 10 times  Classified as having been drunk at least once in their lifetime if they reported any response other than ‘No never’.  0 No lifetime drunkenness  1 Any lifetime drunkenness* | We chose any lifetime drunkenness as a secondary measure of alcohol consumption for sensitivity analysis, following advice from stakeholder consultation.  This measure captures intensity of drinking, which is related to the risk of acute harms.  We used a binary measure of any lifetime drunkenness as stakeholders were interested in capturing a less ‘deviant’ behaviour than weekly alcohol consumption. |
| **Liking school** |  |
| How do you feel about school at present?  1 I like it a lot  2 I like it a bit  3 I don’t like it very much  4 I don’t like it at all* | The relationship between the available school measures and our construct of interest (commitment to school) is somewhat ambiguous. Since our advisory group did not agree on which measures to include, we included liking school instead of pressure from school work in a sensitivity analysis. |
| **Covariates** | **Rationale** |
| **Family affluence scale** |  |
| Does your family own a car, van or truck?  No (0); yes, one (1); yes, two or more (2)  Do you have your own bedroom for yourself?  No (0); yes (1)  During the past 12 months, how many times did you travel away on holiday [vacation] with your family?  Not at all (0); once (1); twice (2); more than twice (3)  How many computers does your family own?  None (0); one (1); two (2); more than two (3)  Responses to these four questions are summed to create a scale ranging from 0 to 9. We recoded this into a three point scale, which has been frequency used in previous analyses of this dataset [16]. This was coded as two dummy variables for analysis, to capture potentially non-linear effects.  1 Low affluence (0-2)  2 Middle affluence (3-5)  3 High affluence (6-9) | We included these dummy variables to understand the relationship between family affluence and the clustering of health-related behaviours. We also wanted to understand the socio-demographic distribution of declines in adolescent drinking. |
| **Participant sex** |  |
| Are you a boy or a girl?  0 Boy  1 Girl | We included this measure to understand the relationship between participant sex and the clustering of health-related behaviours. We also wanted to understand the socio-demographic distribution of declines in adolescent drinking. |

*Asterisks are used to indicate the *most risky/unhealthy* category for each variable. This is used to present results.

# Supplementary Table 2. Descriptive socio-demographic characteristics

|  | **Participants with missing data** | **Participants by survey year** | | | | | | | |
| --- | --- | --- | --- | --- | --- | --- | --- | --- | --- |
|  | **All years** | **2001/02** | | **2005/06** | | **2009/10** | | **2013/14** | |
|  | **N** | **N** | **%** | **N** | **%** | **N** | **%** | **N** | **%** |
| **Sex** | NA |  |  |  |  |  |  |  |  |
| Male |  | 806 | 46 | 709 | 49 | 490 | 48 | 814 | 51 |
| Female |  | 967 | 54 | 742 | 51 | 625 | 52 | 789 | 49 |
| **Family Affluence Scale** | 399 |  |  |  |  |  |  |  |  |
| Low |  | 120 | 7 | 41 | 3 | 42 | 3 | 27 | 2 |
| Middle |  | 882 | 51 | 452 | 33 | 333 | 31 | 393 | 27 |
| High |  | 726 | 42 | 869 | 64 | 626 | 66 | 1032 | 71 |

N = Unweighted number of participants in the Health Behaviours in School-aged Children study. % = Weighted percentage of participants.

# Supplementary Table 3. Selecting number of latent classes using adjusted-BIC

| **Model** | **Adjusted-BIC** | **Change in adjusted-BIC** |
| --- | --- | --- |
| 2 class | 131051.07 |  |
| 3 class | 130107.02 | -944.05 |
| 4 class | 129688.07 | -418.95 |
| 5 class | 129479.78 | -208.29 |
| 6 class | 129368.36 | -111.42 |

# Supplementary Table 4. Item response probabilities for the four class model

|  | **2001/02** | | | | **2005/06** | | | | **2009/10** | | | | **2013/14** | | | |
| --- | --- | --- | --- | --- | --- | --- | --- | --- | --- | --- | --- | --- | --- | --- | --- | --- |
|  | *1** | *2* | *3* | *4* | *1* | *2* | *3* | *4* | *1* | *2* | *3* | *4* | *1* | *2* | *3* | *4* |
| **Proportion of sample** | 38% | 19% | 29% | 14% | 24% | 26% | 33% | 18% | 21% | 26% | 26% | 26% | 17% | 29% | 33% | 21% |
| **Weekly alcohol use** |  |  |  |  |  |  |  |  |  |  |  |  |  |  |  |  |
| Not weekly | 0.290 | 0.520 | 0.837 | 0.599 | 0.295 | 0.823 | 0.876 | 0.607 | 0.538 | 0.916 | 0.950 | 0.775 | 0.713 | 0.935 | 0.979 | 0.959 |
| Weekly | 0.710 | 0.480 | 0.163 | 0.401 | 0.705 | 0.177 | 0.124 | 0.393 | 0.462 | 0.084 | 0.050 | 0.225 | 0.287 | 0.065 | 0.021 | 0.041 |
| **Cigarette smoking** |  |  |  |  |  |  |  |  |  |  |  |  |  |  |  |  |
| Non-smoker | 0.239 | 0.939 | 0.940 | 0.997 | 0.218 | 0.963 | 0.906 | 0.954 | 0.241 | 0.956 | 0.962 | 0.980 | 0.379 | 0.992 | 0.973 | 0.990 |
| Smoker | 0.761 | 0.061 | 0.060 | 0.003 | 0.782 | 0.037 | 0.094 | 0.046 | 0.759 | 0.044 | 0.038 | 0.020 | 0.621 | 0.008 | 0.027 | 0.010 |
| **Sexual activity** |  |  |  |  |  |  |  |  |  |  |  |  |  |  |  |  |
| Never | 0.304 | 0.716 | 0.857 | 0.828 | 0.314 | 0.869 | 0.821 | 0.815 | 0.236 | 0.862 | 0.820 | 0.828 | 0.324 | 0.909 | 0.876 | 0.900 |
| Had intercourse | 0.696 | 0.284 | 0.143 | 0.172 | 0.686 | 0.131 | 0.179 | 0.185 | 0.764 | 0.138 | 0.180 | 0.172 | 0.676 | 0.091 | 0.124 | 0.100 |
| **Lifetime cannabis use** |  |  |  |  |  |  |  |  |  |  |  |  |  |  |  |  |
| Never | 0.144 | 0.763 | 0.964 | 0.821 | 0.268 | 0.957 | 0.922 | 0.806 | 0.224 | 0.909 | 0.944 | 0.907 | 0.125 | 0.941 | 0.975 | 0.896 |
| Any use | 0.856 | 0.237 | 0.036 | 0.179 | 0.732 | 0.043 | 0.078 | 0.194 | 0.776 | 0.091 | 0.056 | 0.093 | 0.875 | 0.059 | 0.025 | 0.104 |
| **Perceived academic achievement** |  |  |  |  |  |  |  |  |  |  |  |  |  |  |  |  |
| Very good | 0.070 | 0.346 | 0.299 | 0.028 | 0.090 | 0.461 | 0.159 | 0.111 | 0.116 | 0.414 | 0.175 | 0.114 | 0.166 | 0.498 | 0.225 | 0.124 |
| Good | 0.341 | 0.537 | 0.456 | 0.422 | 0.307 | 0.478 | 0.487 | 0.543 | 0.411 | 0.460 | 0.509 | 0.554 | 0.426 | 0.457 | 0.496 | 0.458 |
| Average | 0.442 | 0.117 | 0.234 | 0.451 | 0.414 | 0.061 | 0.304 | 0.311 | 0.369 | 0.127 | 0.294 | 0.304 | 0.311 | 0.044 | 0.250 | 0.376 |
| Below average | 0.146 | 0.000 | 0.011 | 0.099 | 0.189 | 0.000 | 0.050 | 0.035 | 0.103 | 0.000 | 0.022 | 0.029 | 0.097 | 0.002 | 0.029 | 0.042 |
| **Pressure from school work** |  |  |  |  |  |  |  |  |  |  |  |  |  |  |  |  |
| Not at all | 0.095 | 0.070 | 0.044 | 0.074 | 0.061 | 0.097 | 0.015 | 0.062 | 0.061 | 0.079 | 0.024 | 0.117 | 0.069 | 0.089 | 0.022 | 0.080 |
| A little | 0.222 | 0.382 | 0.222 | 0.414 | 0.238 | 0.397 | 0.257 | 0.290 | 0.274 | 0.326 | 0.284 | 0.479 | 0.270 | 0.321 | 0.211 | 0.499 |
| Some | 0.299 | 0.353 | 0.334 | 0.286 | 0.273 | 0.280 | 0.257 | 0.431 | 0.297 | 0.306 | 0.295 | 0.311 | 0.265 | 0.356 | 0.312 | 0.366 |
| A lot | 0.383 | 0.195 | 0.400 | 0.226 | 0.428 | 0.226 | 0.471 | 0.217 | 0.368 | 0.289 | 0.397 | 0.093 | 0.396 | 0.234 | 0.455 | 0.055 |
| **Classmate support scale** |  |  |  |  |  |  |  |  |  |  |  |  |  |  |  |  |
| High support | 0.130 | 0.258 | 0.135 | 0.040 | 0.367 | 0.673 | 0.278 | 0.272 | 0.191 | 0.316 | 0.111 | 0.113 | 0.161 | 0.503 | 0.078 | 0.215 |
| - | 0.223 | 0.340 | 0.193 | 0.141 | 0.225 | 0.173 | 0.252 | 0.322 | 0.262 | 0.382 | 0.216 | 0.296 | 0.205 | 0.254 | 0.212 | 0.284 |
| - | 0.357 | 0.306 | 0.383 | 0.494 | 0.246 | 0.115 | 0.247 | 0.315 | 0.315 | 0.201 | 0.353 | 0.420 | 0.273 | 0.190 | 0.315 | 0.347 |
| Low support | 0.290 | 0.095 | 0.289 | 0.325 | 0.162 | 0.039 | 0.224 | 0.091 | 0.233 | 0.102 | 0.319 | 0.172 | 0.361 | 0.053 | 0.395 | 0.154 |
| **Daily use of e-media** |  |  |  |  |  |  |  |  |  |  |  |  |  |  |  |  |
| No daily use | 0.494 | 0.393 | 0.657 | 0.670 | 0.285 | 0.464 | 0.416 | 0.602 | 0.211 | 0.296 | 0.324 | 0.452 | 0.095 | 0.224 | 0.282 | 0.326 |
| Daily use | 0.506 | 0.607 | 0.343 | 0.330 | 0.715 | 0.536 | 0.584 | 0.398 | 0.789 | 0.704 | 0.676 | 0.548 | 0.905 | 0.776 | 0.718 | 0.674 |
| **Ease of communication with parents** |  |  |  |  |  |  |  |  |  |  |  |  |  |  |  |  |
| One easy to talk to | 0.386 | 0.555 | 0.438 | 0.349 | 0.377 | 0.694 | 0.292 | 0.318 | 0.317 | 0.552 | 0.309 | 0.269 | 0.361 | 0.560 | 0.316 | 0.525 |
| None easy to talk to | 0.614 | 0.445 | 0.562 | 0.651 | 0.623 | 0.306 | 0.708 | 0.682 | 0.683 | 0.448 | 0.691 | 0.731 | 0.639 | 0.440 | 0.684 | 0.475 |
| **Exercise** |  |  |  |  |  |  |  |  |  |  |  |  |  |  |  |  |
| High | 0.169 | 0.247 | 0.080 | 0.207 | 0.176 | 0.155 | 0.049 | 0.207 | 0.199 | 0.164 | 0.104 | 0.262 | 0.160 | 0.196 | 0.049 | 0.174 |
| - | 0.204 | 0.313 | 0.192 | 0.304 | 0.219 | 0.310 | 0.116 | 0.381 | 0.232 | 0.333 | 0.180 | 0.261 | 0.195 | 0.285 | 0.182 | 0.319 |
| - | 0.306 | 0.245 | 0.321 | 0.340 | 0.284 | 0.343 | 0.351 | 0.387 | 0.291 | 0.408 | 0.344 | 0.351 | 0.347 | 0.328 | 0.365 | 0.327 |
| Low | 0.322 | 0.196 | 0.407 | 0.149 | 0.321 | 0.191 | 0.484 | 0.025 | 0.279 | 0.095 | 0.372 | 0.126 | 0.298 | 0.191 | 0.404 | 0.181 |
| **Fruit and vegetable consumption index** |  |  |  |  |  |  |  |  |  |  |  |  |  |  |  |  |
| High consumption | 0.108 | 0.351 | 0.198 | 0.066 | 0.269 | 0.477 | 0.253 | 0.214 | 0.210 | 0.488 | 0.274 | 0.139 | 0.245 | 0.467 | 0.214 | 0.122 |
| - | 0.182 | 0.269 | 0.241 | 0.157 | 0.209 | 0.225 | 0.225 | 0.286 | 0.224 | 0.227 | 0.245 | 0.212 | 0.186 | 0.300 | 0.256 | 0.180 |
| - | 0.287 | 0.257 | 0.251 | 0.382 | 0.209 | 0.208 | 0.280 | 0.321 | 0.284 | 0.200 | 0.216 | 0.396 | 0.300 | 0.188 | 0.289 | 0.431 |
| Low consumption | 0.423 | 0.124 | 0.311 | 0.395 | 0.312 | 0.091 | 0.242 | 0.179 | 0.281 | 0.086 | 0.265 | 0.253 | 0.270 | 0.046 | 0.241 | 0.267 |
| **Life satisfaction** |  |  |  |  |  |  |  |  |  |  |  |  |  |  |  |  |
| High | 0.094 | 0.428 | 0.160 | 0.141 | 0.124 | 0.477 | 0.110 | 0.273 | 0.175 | 0.422 | 0.030 | 0.151 | 0.084 | 0.339 | 0.038 | 0.112 |
| - | 0.226 | 0.399 | 0.232 | 0.253 | 0.236 | 0.383 | 0.220 | 0.334 | 0.207 | 0.439 | 0.196 | 0.324 | 0.161 | 0.461 | 0.117 | 0.253 |
| - | 0.242 | 0.173 | 0.223 | 0.327 | 0.218 | 0.138 | 0.224 | 0.298 | 0.245 | 0.139 | 0.266 | 0.356 | 0.219 | 0.139 | 0.231 | 0.412 |
| Low | 0.438 | 0.000 | 0.386 | 0.278 | 0.423 | 0.001 | 0.446 | 0.095 | 0.372 | 0.000 | 0.508 | 0.170 | 0.536 | 0.062 | 0.614 | 0.223 |

* 1: *Overall unhealthy. 2: Overall healthy. 3: Substance abstainers with behavioural risk indicators. 4: Additional class.*

# Supplementary Table 5. Selecting model specification using adjusted-BIC

| **Model** | **Adjusted-BIC** | **Change in adjusted-BIC** |
| --- | --- | --- |
| Fully constrained | 146190.10 |  |
| Semi-constrained | 145809.62 | -380.48 |
| Fully unconstrained | 145301.67 | -507.95 |
| Fully unconstrained – covariate effect allowed to vary over time | 145392.79 | +91.12 |

# Supplementary Table 6. Class membership and item response probabilities for the primary analysis in 2001/02

|  | *Overall unhealthy* | *Substance abstainers with BRIs* | *Overall healthy* |
| --- | --- | --- | --- |
| **Proportion of sample** | 39% | 40% | 21% |
| **Weekly alcohol use** |  |  |  |
| Not weekly | 0.290 | 0.789 | 0.509 |
| Weekly* | 0.710 | 0.211 | 0.491 |
| **Cigarette smoking** |  |  |  |
| Non-smoker | 0.262 | 0.955 | 0.942 |
| Smoker* | 0.738 | 0.045 | 0.058 |
| **Sexual activity** |  |  |  |
| Never | 0.310 | 0.865 | 0.710 |
| Had intercourse* | 0.690 | 0.135 | 0.290 |
| **Lifetime cannabis use** |  |  |  |
| Never | 0.147 | 0.947 | 0.755 |
| Any use* | 0.853 | 0.053 | 0.245 |
| **Perceived academic achievement** |  |  |  |
| Very good | 0.068 | 0.232 | 0.307 |
| Good | 0.340 | 0.451 | 0.526 |
| Average | 0.447 | 0.277 | 0.167 |
| Below average* | 0.144 | 0.039 | 0.000 |
| **Pressure from school work** |  |  |  |
| Not at all | 0.096 | 0.050 | 0.073 |
| A little | 0.227 | 0.278 | 0.379 |
| Some | 0.299 | 0.318 | 0.353 |
| A lot* | 0.377 | 0.355 | 0.195 |
| **Classmate support scale** |  |  |  |
| High support | 0.127 | 0.111 | 0.239 |
| - | 0.221 | 0.178 | 0.323 |
| - | 0.360 | 0.410 | 0.328 |
| Low support* | 0.292 | 0.301 | 0.109 |
| **Daily use of e-media** |  |  |  |
| No daily use | 0.499 | 0.655 | 0.428 |
| Daily use* | 0.501 | 0.345 | 0.572 |
| **Ease of communication with parents** |  |  |  |
| One parent is easy to talk to | 0.383 | 0.410 | 0.548 |
| None easy to talk to* | 0.617 | 0.590 | 0.452 |
| **Exercise** |  |  |  |
| High | 0.167 | 0.110 | 0.257 |
| - | 0.204 | 0.219 | 0.322 |
| - | 0.310 | 0.326 | 0.248 |
| Low* | 0.318 | 0.345 | 0.173 |
| **Fruit and vegetable consumption index** |  |  |  |
| High consumption | 0.105 | 0.162 | 0.330 |
| - | 0.179 | 0.223 | 0.253 |
| - | 0.288 | 0.286 | 0.271 |
| Low consumption* | 0.427 | 0.329 | 0.146 |
| **Life satisfaction** |  |  |  |
| High | 0.095 | 0.137 | 0.440 |
| - | 0.223 | 0.246 | 0.377 |
| - | 0.246 | 0.251 | 0.183 |
| Low* | 0.436 | 0.366 | 0.000 |

BRIs: behavioural risk indicators. *Asterisks are used to indicate the most risky/unhealthy category for each variable. This is used to present results.

# Supplementary Table 7. Item response probabilities for the primary analysis

|  | **Overall unhealthy** | | | | **Substance abstainers with behavioural risk indicators** | | | | **Overall healthy** | | | |
| --- | --- | --- | --- | --- | --- | --- | --- | --- | --- | --- | --- | --- |
|  | 2001/02 | 2005/06 | 2009/10 | 2013/14 | 2001/02 | 2005/06 | 2009/10 | 2013/14 | 2001/02 | 2005/06 | 2009/10 | 2013/14 |
| **Proportion of sample** | 39% | 26% | 22% | 18% | 40% | 34% | 41% | 41% | 21% | 39% | 37% | 41% |
| **Weekly alcohol use** |  |  |  |  |  |  |  |  |  |  |  |  |
| Not weekly | 0.29 | 0.309 | 0.538 | 0.718 | 0.789 | 0.853 | 0.915 | 0.978 | 0.509 | 0.768 | 0.848 | 0.941 |
| Weekly | 0.71 | 0.691 | 0.462 | 0.282 | 0.211 | 0.147 | 0.085 | 0.022 | 0.491 | 0.232 | 0.152 | 0.059 |
| **Cigarette smoking** |  |  |  |  |  |  |  |  |  |  |  |  |
| Non-smoker | 0.262 | 0.27 | 0.249 | 0.394 | 0.955 | 0.929 | 0.974 | 0.977 | 0.942 | 0.957 | 0.964 | 0.995 |
| Smoker | 0.738 | 0.73 | 0.751 | 0.606 | 0.045 | 0.071 | 0.026 | 0.023 | 0.058 | 0.043 | 0.036 | 0.005 |
| **Sexual activity** |  |  |  |  |  |  |  |  |  |  |  |  |
| Never | 0.31 | 0.336 | 0.25 | 0.328 | 0.865 | 0.852 | 0.833 | 0.884 | 0.71 | 0.842 | 0.844 | 0.91 |
| Had intercourse | 0.69 | 0.664 | 0.75 | 0.672 | 0.135 | 0.148 | 0.167 | 0.116 | 0.29 | 0.158 | 0.156 | 0.09 |
| **Lifetime cannabis use** |  |  |  |  |  |  |  |  |  |  |  |  |
| Never | 0.147 | 0.294 | 0.239 | 0.14 | 0.947 | 0.932 | 0.933 | 0.962 | 0.755 | 0.909 | 0.91 | 0.93 |
| Any use | 0.853 | 0.706 | 0.761 | 0.86 | 0.053 | 0.068 | 0.067 | 0.038 | 0.245 | 0.091 | 0.09 | 0.07 |
| **Perceived academic achievement** |  |  |  |  |  |  |  |  |  |  |  |  |
| Very good | 0.068 | 0.082 | 0.112 | 0.162 | 0.232 | 0.148 | 0.151 | 0.196 | 0.307 | 0.354 | 0.329 | 0.398 |
| Good | 0.34 | 0.315 | 0.405 | 0.431 | 0.451 | 0.484 | 0.512 | 0.469 | 0.526 | 0.517 | 0.508 | 0.475 |
| Average | 0.447 | 0.425 | 0.38 | 0.316 | 0.277 | 0.317 | 0.307 | 0.295 | 0.167 | 0.121 | 0.162 | 0.121 |
| Below average | 0.144 | 0.178 | 0.103 | 0.092 | 0.039 | 0.051 | 0.03 | 0.039 | 0 | 0.008 | 0.001 | 0.007 |
| **Pressure from school work** |  |  |  |  |  |  |  |  |  |  |  |  |
| Not at all | 0.096 | 0.058 | 0.067 | 0.07 | 0.05 | 0.016 | 0.049 | 0.033 | 0.073 | 0.088 | 0.096 | 0.086 |
| A little | 0.227 | 0.24 | 0.273 | 0.281 | 0.278 | 0.255 | 0.358 | 0.282 | 0.379 | 0.366 | 0.371 | 0.361 |
| Some | 0.299 | 0.282 | 0.294 | 0.265 | 0.318 | 0.278 | 0.309 | 0.32 | 0.353 | 0.326 | 0.3 | 0.364 |
| A lot | 0.377 | 0.419 | 0.366 | 0.383 | 0.355 | 0.451 | 0.284 | 0.365 | 0.195 | 0.22 | 0.233 | 0.189 |
| **Classmate support scale** |  |  |  |  |  |  |  |  |  |  |  |  |
| High support | 0.127 | 0.347 | 0.192 | 0.161 | 0.111 | 0.254 | 0.099 | 0.084 | 0.239 | 0.562 | 0.268 | 0.442 |
| - | 0.221 | 0.233 | 0.26 | 0.206 | 0.178 | 0.243 | 0.238 | 0.217 | 0.323 | 0.236 | 0.366 | 0.273 |
| - | 0.36 | 0.261 | 0.317 | 0.282 | 0.41 | 0.264 | 0.382 | 0.322 | 0.328 | 0.166 | 0.259 | 0.232 |
| Low support | 0.292 | 0.159 | 0.231 | 0.351 | 0.301 | 0.239 | 0.281 | 0.376 | 0.109 | 0.035 | 0.107 | 0.053 |
| **Daily use of e-media** |  |  |  |  |  |  |  |  |  |  |  |  |
| No daily use | 0.499 | 0.295 | 0.218 | 0.097 | 0.655 | 0.457 | 0.367 | 0.304 | 0.428 | 0.501 | 0.344 | 0.243 |
| Daily use | 0.501 | 0.705 | 0.782 | 0.903 | 0.345 | 0.543 | 0.633 | 0.696 | 0.572 | 0.499 | 0.656 | 0.757 |
| **Ease of communication with parents** |  |  |  |  |  |  |  |  |  |  |  |  |
| One easy to talk to | 0.383 | 0.363 | 0.321 | 0.366 | 0.41 | 0.288 | 0.288 | 0.341 | 0.548 | 0.574 | 0.474 | 0.567 |
| None easy to talk to | 0.617 | 0.637 | 0.679 | 0.634 | 0.59 | 0.712 | 0.712 | 0.659 | 0.452 | 0.426 | 0.526 | 0.433 |
| **Exercise** |  |  |  |  |  |  |  |  |  |  |  |  |
| High | 0.167 | 0.171 | 0.197 | 0.163 | 0.11 | 0.073 | 0.156 | 0.066 | 0.257 | 0.164 | 0.201 | 0.197 |
| - | 0.204 | 0.229 | 0.235 | 0.199 | 0.219 | 0.145 | 0.192 | 0.207 | 0.322 | 0.326 | 0.329 | 0.298 |
| - | 0.31 | 0.288 | 0.289 | 0.348 | 0.326 | 0.361 | 0.349 | 0.35 | 0.248 | 0.356 | 0.39 | 0.334 |
| Low | 0.318 | 0.312 | 0.279 | 0.291 | 0.345 | 0.421 | 0.303 | 0.376 | 0.173 | 0.154 | 0.081 | 0.171 |
| **Fruit and vegetable consumption index** |  |  |  |  |  |  |  |  |  |  |  |  |
| High consumption | 0.105 | 0.258 | 0.21 | 0.24 | 0.162 | 0.219 | 0.221 | 0.182 | 0.33 | 0.418 | 0.387 | 0.379 |
| - | 0.179 | 0.218 | 0.219 | 0.186 | 0.223 | 0.234 | 0.224 | 0.231 | 0.253 | 0.239 | 0.235 | 0.274 |
| - | 0.288 | 0.217 | 0.282 | 0.3 | 0.286 | 0.301 | 0.284 | 0.321 | 0.271 | 0.233 | 0.256 | 0.258 |
| Low consumption | 0.427 | 0.306 | 0.289 | 0.274 | 0.329 | 0.246 | 0.27 | 0.266 | 0.146 | 0.11 | 0.121 | 0.089 |
| **Life satisfaction** |  |  |  |  |  |  |  |  |  |  |  |  |
| High | 0.095 | 0.124 | 0.173 | 0.084 | 0.137 | 0.101 | 0.05 | 0.039 | 0.44 | 0.431 | 0.367 | 0.287 |
| - | 0.223 | 0.229 | 0.206 | 0.162 | 0.246 | 0.208 | 0.219 | 0.121 | 0.377 | 0.393 | 0.433 | 0.426 |
| - | 0.246 | 0.231 | 0.25 | 0.227 | 0.251 | 0.244 | 0.306 | 0.274 | 0.183 | 0.176 | 0.199 | 0.212 |
| Low | 0.436 | 0.416 | 0.371 | 0.528 | 0.366 | 0.446 | 0.425 | 0.566 | 0 | 0 | 0 | 0.075 |

# Supplementary Table 8. Item response probabilities for the lifetime drunkenness sensitivity analysis

|  | **2001/02** | | | **2005/06** | | | **2009/10** | | | **2013/14** | | |
| --- | --- | --- | --- | --- | --- | --- | --- | --- | --- | --- | --- | --- |
|  | *1** | *2* | *3* | *1* | *2* | *3* | *1* | *2* | *3* | *1* | *2* | *3* |
| **Proportion of sample** | 42% | 37% | 21% | 35% | 29% | 37% | 32% | 43% | 24% | 23% | 37% | 40% |
| **Lifetime drunkenness** |  |  |  |  |  |  |  |  |  |  |  |  |
| None | 0.032 | 0.601 | 0.287 | 0.045 | 0.571 | 0.525 | 0.053 | 0.638 | 0.634 | 0.089 | 0.757 | 0.696 |
| Any | 0.968 | 0.399 | 0.713 | 0.955 | 0.429 | 0.475 | 0.947 | 0.362 | 0.366 | 0.911 | 0.243 | 0.304 |
| **Cigarette smoking** |  |  |  |  |  |  |  |  |  |  |  |  |
| Non-smoker | 0.291 | 0.958 | 0.958 | 0.384 | 0.976 | 0.967 | 0.443 | 0.991 | 0.981 | 0.505 | 0.990 | 0.996 |
| Smoker | 0.709 | 0.042 | 0.042 | 0.616 | 0.024 | 0.033 | 0.557 | 0.009 | 0.019 | 0.495 | 0.010 | 0.004 |
| **Sexual activity** |  |  |  |  |  |  |  |  |  |  |  |  |
| Never | 0.316 | 0.900 | 0.704 | 0.378 | 0.919 | 0.866 | 0.333 | 0.869 | 0.923 | 0.361 | 0.923 | 0.928 |
| Had intercourse | 0.684 | 0.100 | 0.296 | 0.622 | 0.081 | 0.134 | 0.667 | 0.131 | 0.077 | 0.639 | 0.077 | 0.072 |
| **Lifetime cannabis use** |  |  |  |  |  |  |  |  |  |  |  |  |
| Never | 0.175 | 0.961 | 0.774 | 0.399 | 0.969 | 0.924 | 0.367 | 0.970 | 0.957 | 0.266 | 0.978 | 0.949 |
| Any use | 0.825 | 0.039 | 0.226 | 0.601 | 0.031 | 0.076 | 0.633 | 0.030 | 0.043 | 0.734 | 0.022 | 0.051 |
| **Perceived academic achievement** |  |  |  |  |  |  |  |  |  |  |  |  |
| Very good | 0.070 | 0.246 | 0.297 | 0.086 | 0.158 | 0.373 | 0.126 | 0.153 | 0.415 | 0.181 | 0.195 | 0.399 |
| Good | 0.347 | 0.446 | 0.534 | 0.356 | 0.487 | 0.517 | 0.427 | 0.549 | 0.457 | 0.447 | 0.463 | 0.477 |
| Average | 0.446 | 0.268 | 0.169 | 0.413 | 0.302 | 0.107 | 0.368 | 0.276 | 0.127 | 0.299 | 0.300 | 0.118 |
| Below average | 0.138 | 0.040 | 0.000 | 0.145 | 0.053 | 0.003 | 0.079 | 0.023 | 0.001 | 0.074 | 0.043 | 0.006 |
| **Pressure from school work** |  |  |  |  |  |  |  |  |  |  |  |  |
| Not at all | 0.092 | 0.049 | 0.077 | 0.050 | 0.017 | 0.091 | 0.073 | 0.057 | 0.091 | 0.062 | 0.037 | 0.084 |
| A little | 0.233 | 0.275 | 0.376 | 0.241 | 0.259 | 0.373 | 0.287 | 0.388 | 0.342 | 0.283 | 0.286 | 0.359 |
| Some | 0.299 | 0.317 | 0.355 | 0.280 | 0.291 | 0.320 | 0.296 | 0.299 | 0.316 | 0.273 | 0.324 | 0.364 |
| A lot | 0.376 | 0.359 | 0.191 | 0.429 | 0.432 | 0.216 | 0.343 | 0.255 | 0.251 | 0.382 | 0.352 | 0.193 |
| **Classmate support scale** |  |  |  |  |  |  |  |  |  |  |  |  |
| High support | 0.130 | 0.104 | 0.242 | 0.348 | 0.240 | 0.573 | 0.182 | 0.102 | 0.322 | 0.168 | 0.078 | 0.445 |
| - | 0.223 | 0.168 | 0.332 | 0.243 | 0.234 | 0.236 | 0.270 | 0.245 | 0.396 | 0.198 | 0.225 | 0.274 |
| - | 0.363 | 0.411 | 0.329 | 0.246 | 0.283 | 0.158 | 0.329 | 0.398 | 0.182 | 0.283 | 0.324 | 0.233 |
| Low support | 0.285 | 0.318 | 0.097 | 0.162 | 0.243 | 0.033 | 0.219 | 0.255 | 0.100 | 0.351 | 0.373 | 0.049 |
| **Daily use of e-media** |  |  |  |  |  |  |  |  |  |  |  |  |
| No daily use | 0.498 | 0.666 | 0.431 | 0.298 | 0.501 | 0.504 | 0.198 | 0.399 | 0.366 | 0.110 | 0.322 | 0.244 |
| Daily use | 0.502 | 0.334 | 0.569 | 0.702 | 0.499 | 0.496 | 0.802 | 0.601 | 0.634 | 0.890 | 0.678 | 0.756 |
| **Ease of communication with parents** |  |  |  |  |  |  |  |  |  |  |  |  |
| One easy to talk to | 0.392 | 0.400 | 0.550 | 0.354 | 0.277 | 0.594 | 0.316 | 0.301 | 0.539 | 0.354 | 0.356 | 0.565 |
| None easy to talk to | 0.608 | 0.600 | 0.450 | 0.646 | 0.723 | 0.406 | 0.684 | 0.699 | 0.461 | 0.646 | 0.644 | 0.435 |
| **Exercise** |  |  |  |  |  |  |  |  |  |  |  |  |
| High | 0.170 | 0.109 | 0.245 | 0.154 | 0.076 | 0.162 | 0.180 | 0.185 | 0.178 | 0.156 | 0.066 | 0.194 |
| - | 0.207 | 0.222 | 0.310 | 0.211 | 0.160 | 0.325 | 0.251 | 0.197 | 0.352 | 0.213 | 0.195 | 0.305 |
| - | 0.310 | 0.322 | 0.256 | 0.312 | 0.364 | 0.346 | 0.312 | 0.359 | 0.388 | 0.361 | 0.348 | 0.328 |
| Low | 0.313 | 0.346 | 0.188 | 0.322 | 0.399 | 0.167 | 0.257 | 0.259 | 0.082 | 0.270 | 0.391 | 0.173 |
| **Fruit and vegetable consumption index** |  |  |  |  |  |  |  |  |  |  |  |  |
| High consumption | 0.104 | 0.181 | 0.302 | 0.255 | 0.207 | 0.435 | 0.242 | 0.198 | 0.477 | 0.242 | 0.173 | 0.386 |
| - | 0.185 | 0.214 | 0.261 | 0.216 | 0.255 | 0.229 | 0.220 | 0.222 | 0.245 | 0.194 | 0.231 | 0.276 |
| - | 0.283 | 0.291 | 0.272 | 0.230 | 0.311 | 0.228 | 0.274 | 0.324 | 0.184 | 0.317 | 0.315 | 0.254 |
| Low consumption | 0.428 | 0.313 | 0.164 | 0.299 | 0.227 | 0.108 | 0.264 | 0.256 | 0.094 | 0.247 | 0.281 | 0.083 |
| **Life satisfaction** |  |  |  |  |  |  |  |  |  |  |  |  |
| High | 0.100 | 0.144 | 0.416 | 0.131 | 0.109 | 0.437 | 0.182 | 0.071 | 0.429 | 0.083 | 0.043 | 0.286 |
| - | 0.232 | 0.226 | 0.395 | 0.242 | 0.206 | 0.391 | 0.242 | 0.268 | 0.413 | 0.184 | 0.113 | 0.426 |
| - | 0.247 | 0.247 | 0.188 | 0.239 | 0.238 | 0.172 | 0.247 | 0.313 | 0.157 | 0.237 | 0.275 | 0.209 |
| Low | 0.420 | 0.383 | 0.000 | 0.388 | 0.448 | 0.000 | 0.329 | 0.348 | 0.001 | 0.496 | 0.569 | 0.079 |

* 1: *Overall unhealthy. 2: Substance abstainers with behavioural risk indicators. 3: Overall healthy.*

# Supplementary Table 9. Item response probabilities for the weekly drinking with alcopops sensitivity analysis

|  | **2001/02** | | | **2005/06** | | | **2009/10** | | | **2013/14** | | |
| --- | --- | --- | --- | --- | --- | --- | --- | --- | --- | --- | --- | --- |
|  | *1** | *2* | *3* | *1* | *2* | *3* | *1* | *2* | *3* | *1* | *2* | *3* |
| **Proportion of sample** | 39% | 39% | 22% | 26% | 34% | 40% | 23% | 37% | 40% | 17% | 41% | 41% |
| **Weekly alcohol use** |  |  |  |  |  |  |  |  |  |  |  |  |
| Not weekly | 0.296 | 0.793 | 0.529 | 0.235 | 0.814 | 0.736 | 0.453 | 0.897 | 0.837 | 0.706 | 0.974 | 0.934 |
| Weekly | 0.704 | 0.207 | 0.471 | 0.765 | 0.186 | 0.264 | 0.547 | 0.103 | 0.163 | 0.294 | 0.026 | 0.066 |
| **Cigarette smoking** |  |  |  |  |  |  |  |  |  |  |  |  |
| Non-smoker | 0.263 | 0.953 | 0.946 | 0.281 | 0.925 | 0.954 | 0.279 | 0.973 | 0.966 | 0.393 | 0.975 | 0.995 |
| Smoker | 0.737 | 0.047 | 0.054 | 0.719 | 0.075 | 0.046 | 0.721 | 0.027 | 0.034 | 0.607 | 0.025 | 0.005 |
| **Sexual activity** |  |  |  |  |  |  |  |  |  |  |  |  |
| Never | 0.309 | 0.865 | 0.716 | 0.331 | 0.854 | 0.843 | 0.255 | 0.851 | 0.839 | 0.327 | 0.881 | 0.911 |
| Had intercourse | 0.691 | 0.135 | 0.284 | 0.669 | 0.146 | 0.157 | 0.745 | 0.149 | 0.161 | 0.673 | 0.119 | 0.089 |
| **Lifetime cannabis use** |  |  |  |  |  |  |  |  |  |  |  |  |
| Never | 0.146 | 0.949 | 0.763 | 0.297 | 0.929 | 0.909 | 0.256 | 0.936 | 0.916 | 0.132 | 0.962 | 0.931 |
| Any use | 0.854 | 0.051 | 0.237 | 0.703 | 0.071 | 0.091 | 0.744 | 0.064 | 0.084 | 0.868 | 0.038 | 0.069 |
| **Perceived academic achievement** |  |  |  |  |  |  |  |  |  |  |  |  |
| Very good | 0.069 | 0.230 | 0.306 | 0.078 | 0.150 | 0.352 | 0.113 | 0.148 | 0.319 | 0.161 | 0.195 | 0.398 |
| Good | 0.340 | 0.449 | 0.527 | 0.315 | 0.482 | 0.518 | 0.403 | 0.511 | 0.512 | 0.431 | 0.469 | 0.475 |
| Average | 0.446 | 0.280 | 0.167 | 0.430 | 0.316 | 0.122 | 0.384 | 0.310 | 0.167 | 0.316 | 0.297 | 0.120 |
| Below average | 0.144 | 0.040 | 0.000 | 0.177 | 0.051 | 0.008 | 0.100 | 0.031 | 0.002 | 0.092 | 0.039 | 0.007 |
| **Pressure from school work** |  |  |  |  |  |  |  |  |  |  |  |  |
| Not at all | 0.095 | 0.051 | 0.071 | 0.057 | 0.017 | 0.087 | 0.066 | 0.048 | 0.093 | 0.070 | 0.033 | 0.086 |
| A little | 0.228 | 0.276 | 0.377 | 0.238 | 0.255 | 0.366 | 0.281 | 0.347 | 0.376 | 0.280 | 0.283 | 0.361 |
| Some | 0.300 | 0.315 | 0.355 | 0.283 | 0.278 | 0.325 | 0.284 | 0.314 | 0.302 | 0.269 | 0.319 | 0.363 |
| A lot | 0.377 | 0.358 | 0.196 | 0.422 | 0.451 | 0.222 | 0.368 | 0.291 | 0.229 | 0.382 | 0.365 | 0.190 |
| **Classmate support scale** |  |  |  |  |  |  |  |  |  |  |  |  |
| High support | 0.127 | 0.111 | 0.236 | 0.340 | 0.257 | 0.560 | 0.189 | 0.097 | 0.256 | 0.161 | 0.082 | 0.442 |
| - | 0.222 | 0.176 | 0.322 | 0.233 | 0.242 | 0.237 | 0.253 | 0.237 | 0.361 | 0.206 | 0.217 | 0.273 |
| - | 0.360 | 0.409 | 0.332 | 0.268 | 0.258 | 0.168 | 0.319 | 0.380 | 0.269 | 0.284 | 0.322 | 0.232 |
| Low support | 0.292 | 0.304 | 0.110 | 0.159 | 0.242 | 0.035 | 0.239 | 0.285 | 0.114 | 0.349 | 0.378 | 0.053 |
| **Daily use of e-media** |  |  |  |  |  |  |  |  |  |  |  |  |
| No daily use | 0.498 | 0.657 | 0.435 | 0.286 | 0.465 | 0.499 | 0.209 | 0.378 | 0.344 | 0.098 | 0.303 | 0.243 |
| Daily use | 0.502 | 0.343 | 0.565 | 0.714 | 0.535 | 0.501 | 0.791 | 0.622 | 0.656 | 0.902 | 0.697 | 0.757 |
| **Ease of communication with parents** |  |  |  |  |  |  |  |  |  |  |  |  |
| One easy to talk to | 0.384 | 0.409 | 0.544 | 0.361 | 0.285 | 0.573 | 0.328 | 0.281 | 0.462 | 0.369 | 0.341 | 0.566 |
| None easy to talk to | 0.616 | 0.591 | 0.456 | 0.639 | 0.715 | 0.427 | 0.672 | 0.719 | 0.538 | 0.631 | 0.659 | 0.434 |
| **Exercise** |  |  |  |  |  |  |  |  |  |  |  |  |
| High | 0.168 | 0.109 | 0.252 | 0.168 | 0.075 | 0.163 | 0.191 | 0.152 | 0.201 | 0.164 | 0.066 | 0.197 |
| - | 0.205 | 0.218 | 0.320 | 0.233 | 0.140 | 0.324 | 0.236 | 0.190 | 0.318 | 0.199 | 0.207 | 0.297 |
| - | 0.309 | 0.327 | 0.251 | 0.286 | 0.363 | 0.356 | 0.296 | 0.337 | 0.395 | 0.348 | 0.351 | 0.333 |
| Low | 0.319 | 0.346 | 0.177 | 0.313 | 0.422 | 0.157 | 0.277 | 0.321 | 0.086 | 0.289 | 0.376 | 0.172 |
| **Fruit and vegetable consumption index** |  |  |  |  |  |  |  |  |  |  |  |  |
| High consumption | 0.106 | 0.161 | 0.326 | 0.255 | 0.220 | 0.416 | 0.214 | 0.221 | 0.375 | 0.241 | 0.181 | 0.379 |
| - | 0.180 | 0.222 | 0.253 | 0.220 | 0.232 | 0.240 | 0.226 | 0.221 | 0.235 | 0.185 | 0.231 | 0.275 |
| - | 0.288 | 0.286 | 0.272 | 0.219 | 0.301 | 0.233 | 0.276 | 0.279 | 0.264 | 0.300 | 0.321 | 0.257 |
| Low consumption | 0.426 | 0.331 | 0.150 | 0.306 | 0.246 | 0.112 | 0.284 | 0.280 | 0.127 | 0.274 | 0.267 | 0.089 |
| **Life satisfaction** |  |  |  |  |  |  |  |  |  |  |  |  |
| High | 0.096 | 0.133 | 0.434 | 0.123 | 0.099 | 0.429 | 0.172 | 0.040 | 0.349 | 0.085 | 0.039 | 0.286 |
| - | 0.224 | 0.242 | 0.379 | 0.234 | 0.198 | 0.395 | 0.211 | 0.200 | 0.432 | 0.164 | 0.119 | 0.425 |
| - | 0.245 | 0.250 | 0.186 | 0.230 | 0.247 | 0.176 | 0.253 | 0.293 | 0.218 | 0.226 | 0.275 | 0.212 |
| Low | 0.435 | 0.375 | 0.000 | 0.413 | 0.457 | 0.000 | 0.363 | 0.466 | 0.001 | 0.526 | 0.567 | 0.077 |

* 1: *Overall unhealthy. 2: Substance abstainers with behavioural risk indicators. 3: Overall healthy.*

# Supplementary Table 10. Item response probabilities for the liking school sensitivity analysis

|  | **2001/02** | | | **2005/06** | | | **2009/10** | | | **2013/14** | | |
| --- | --- | --- | --- | --- | --- | --- | --- | --- | --- | --- | --- | --- |
|  | *1** | *2* | *3* | *1* | *2* | *3* | *1* | *2* | *3* | *1* | *2* | *3* |
| **Proportion of sample** | 38% | 31% | 31% | 26% | 28% | 46% | 22% | 48% | 31% | 17% | 44% | 38% |
| **Weekly alcohol use** |  |  |  |  |  |  |  |  |  |  |  |  |
| Not weekly | 0.278 | 0.699 | 0.691 | 0.317 | 0.852 | 0.776 | 0.54 | 0.891 | 0.864 | 0.717 | 0.974 | 0.941 |
| Weekly | 0.722 | 0.301 | 0.309 | 0.683 | 0.148 | 0.224 | 0.46 | 0.109 | 0.136 | 0.283 | 0.026 | 0.059 |
| **Cigarette smoking** |  |  |  |  |  |  |  |  |  |  |  |  |
| Non-smoker | 0.254 | 0.951 | 0.94 | 0.284 | 0.929 | 0.944 | 0.255 | 0.978 | 0.943 | 0.391 | 0.977 | 0.993 |
| Smoker | 0.746 | 0.049 | 0.06 | 0.716 | 0.071 | 0.056 | 0.745 | 0.022 | 0.057 | 0.609 | 0.023 | 0.007 |
| **Sexual activity** |  |  |  |  |  |  |  |  |  |  |  |  |
| Never | 0.292 | 0.811 | 0.823 | 0.331 | 0.854 | 0.844 | 0.247 | 0.821 | 0.857 | 0.333 | 0.888 | 0.902 |
| Had intercourse | 0.708 | 0.189 | 0.177 | 0.669 | 0.146 | 0.156 | 0.753 | 0.179 | 0.143 | 0.667 | 0.112 | 0.098 |
| **Lifetime cannabis use** |  |  |  |  |  |  |  |  |  |  |  |  |
| Never | 0.137 | 0.923 | 0.836 | 0.294 | 0.935 | 0.909 | 0.235 | 0.938 | 0.891 | 0.141 | 0.951 | 0.934 |
| Any use | 0.863 | 0.077 | 0.164 | 0.706 | 0.065 | 0.091 | 0.765 | 0.062 | 0.109 | 0.859 | 0.049 | 0.066 |
| **Perceived academic achievement** |  |  |  |  |  |  |  |  |  |  |  |  |
| Very good | 0.064 | 0.125 | 0.39 | 0.074 | 0.092 | 0.363 | 0.095 | 0.147 | 0.384 | 0.167 | 0.176 | 0.433 |
| Good | 0.342 | 0.427 | 0.521 | 0.309 | 0.453 | 0.534 | 0.407 | 0.518 | 0.496 | 0.416 | 0.463 | 0.488 |
| Average | 0.45 | 0.392 | 0.089 | 0.436 | 0.392 | 0.099 | 0.394 | 0.309 | 0.119 | 0.321 | 0.323 | 0.074 |
| Below average | 0.144 | 0.056 | 0 | 0.182 | 0.064 | 0.004 | 0.103 | 0.026 | 0.002 | 0.095 | 0.037 | 0.006 |
| **Liking school** |  |  |  |  |  |  |  |  |  |  |  |  |
| A lot | 0.064 | 0.013 | 0.398 | 0.107 | 0.034 | 0.458 | 0.051 | 0.073 | 0.343 | 0.085 | 0.039 | 0.417 |
| A bit | 0.361 | 0.463 | 0.511 | 0.41 | 0.543 | 0.523 | 0.385 | 0.592 | 0.622 | 0.413 | 0.579 | 0.519 |
| Not very much | 0.3 | 0.406 | 0.078 | 0.279 | 0.314 | 0.009 | 0.319 | 0.233 | 0.034 | 0.279 | 0.297 | 0.048 |
| Not at all | 0.276 | 0.118 | 0.013 | 0.204 | 0.109 | 0.01 | 0.245 | 0.103 | 0 | 0.223 | 0.085 | 0.017 |
| **Classmate support scale** |  |  |  |  |  |  |  |  |  |  |  |  |
| High support | 0.136 | 0.048 | 0.25 | 0.345 | 0.165 | 0.572 | 0.171 | 0.105 | 0.308 | 0.16 | 0.075 | 0.48 |
| - | 0.222 | 0.148 | 0.307 | 0.232 | 0.238 | 0.241 | 0.262 | 0.234 | 0.396 | 0.201 | 0.234 | 0.26 |
| - | 0.357 | 0.429 | 0.338 | 0.263 | 0.305 | 0.154 | 0.326 | 0.393 | 0.213 | 0.275 | 0.332 | 0.217 |
| Low support | 0.285 | 0.375 | 0.105 | 0.161 | 0.291 | 0.032 | 0.242 | 0.268 | 0.083 | 0.364 | 0.358 | 0.043 |
| **Daily use of e-media** |  |  |  |  |  |  |  |  |  |  |  |  |
| No daily use | 0.488 | 0.631 | 0.535 | 0.291 | 0.49 | 0.476 | 0.217 | 0.361 | 0.348 | 0.09 | 0.307 | 0.238 |
| Daily use | 0.512 | 0.369 | 0.465 | 0.709 | 0.51 | 0.524 | 0.783 | 0.639 | 0.652 | 0.91 | 0.693 | 0.762 |
| **Ease of communication with parents** |  |  |  |  |  |  |  |  |  |  |  |  |
| One easy to talk to | 0.395 | 0.382 | 0.517 | 0.359 | 0.291 | 0.533 | 0.311 | 0.295 | 0.508 | 0.365 | 0.364 | 0.557 |
| None easy to talk to | 0.605 | 0.618 | 0.483 | 0.641 | 0.709 | 0.467 | 0.689 | 0.705 | 0.492 | 0.635 | 0.636 | 0.443 |
| **Exercise** |  |  |  |  |  |  |  |  |  |  |  |  |
| High | 0.181 | 0.135 | 0.169 | 0.168 | 0.08 | 0.149 | 0.198 | 0.179 | 0.175 | 0.162 | 0.087 | 0.185 |
| - | 0.207 | 0.243 | 0.262 | 0.224 | 0.153 | 0.297 | 0.226 | 0.207 | 0.341 | 0.193 | 0.214 | 0.299 |
| - | 0.302 | 0.316 | 0.291 | 0.289 | 0.373 | 0.349 | 0.297 | 0.343 | 0.401 | 0.348 | 0.359 | 0.322 |
| Low | 0.31 | 0.306 | 0.278 | 0.318 | 0.395 | 0.205 | 0.279 | 0.271 | 0.084 | 0.297 | 0.339 | 0.194 |
| **Fruit and vegetable consumption index** |  |  |  |  |  |  |  |  |  |  |  |  |
| High consumption | 0.114 | 0.12 | 0.305 | 0.258 | 0.152 | 0.429 | 0.209 | 0.213 | 0.435 | 0.233 | 0.171 | 0.41 |
| - | 0.181 | 0.22 | 0.243 | 0.217 | 0.252 | 0.229 | 0.219 | 0.228 | 0.231 | 0.192 | 0.226 | 0.28 |
| - | 0.286 | 0.314 | 0.25 | 0.216 | 0.328 | 0.227 | 0.282 | 0.298 | 0.23 | 0.297 | 0.337 | 0.236 |
| Low consumption | 0.419 | 0.346 | 0.202 | 0.309 | 0.269 | 0.114 | 0.29 | 0.26 | 0.105 | 0.278 | 0.266 | 0.075 |
| **Life satisfaction** |  |  |  |  |  |  |  |  |  |  |  |  |
| High | 0.109 | 0.126 | 0.335 | 0.124 | 0.118 | 0.373 | 0.159 | 0.071 | 0.414 | 0.086 | 0.048 | 0.295 |
| - | 0.232 | 0.183 | 0.387 | 0.223 | 0.218 | 0.364 | 0.207 | 0.248 | 0.431 | 0.162 | 0.15 | 0.415 |
| - | 0.24 | 0.286 | 0.177 | 0.229 | 0.231 | 0.195 | 0.248 | 0.318 | 0.154 | 0.219 | 0.29 | 0.192 |
| Low | 0.419 | 0.406 | 0.102 | 0.424 | 0.433 | 0.068 | 0.386 | 0.363 | 0.001 | 0.533 | 0.511 | 0.099 |

* 1: *Overall unhealthy. 2: Substance abstainers with behavioural risk indicators. 3: Overall healthy.*
